# Supplementary material for: Differential effect of supercoiling on bacterial transcription in topological domains
Source: PLoS Comput Biol. 2025 Nov 11;21(11):e1012764. doi: 10.1371/journal.pcbi.1012764 (PMC12622846; doi:10.1371/journal.pcbi.1012764)
Supplement: S1 Table — (DOCX) [file pcbi.1012764.s001.docx]

**S1 Table. Summary of several Published Models of Bacterial SC-Coupled Transcription**

| Primary Author | Year | SC Diffusion | Initiation | Elongation | RNAP rotation | mRNA | Topoisomerases | Experimental comparisons |
| --- | --- | --- | --- | --- | --- | --- | --- | --- |
| Sevier [1] | 2018 | Instant. | 1 stage. Constant rate. | Slows with sigmoidal functions of upstream and downstream torque from [2]. | Yes, according to a physical model of RNAP drag. | Yes; decay exponentially. | Stochastically restore DNA between two genes to relaxed state. | None. |
| El Houdaigui [3] | 2019 | Instant. | 1 stage. Sigmoidal SC-dependency. | Constant rate. | Rotates by constant proportion of passed DNA rotations. | No. | Deterministic activity of TopoI and gyrase. Sigmoidal SC-dependencies. | Expression data and CHIP-Seq for topoisomerases [4-8]. |
| Ancona [9] | 2019 | Finite diffusion on lattice. | 1 stage. Linear SC-dependency. | Constant rate. | Decreases with nascent mRNA length as transcription proceeds. | No. | Stochastically restore DNA to relaxed state. | None. |
| Tripathi [10] | 2022 | Instant. | 1 stage. Constant rate. | Slows with sigmoidal dependency on net torque. | Yes, according to physical model of RNAP drag. | No. | Stochastically restore DNA to relaxed state. | Data relating initiation and elongation rates [11]. |
| Geng [12] | 2022 | Biased Random-walk on lattice. | 1 stage. Piecewise linear SC-dependency. | Stalls under sufficient torque from [2]. | Stochastically rotates at some constant rate. | No. | TopoI and gyrase are discrete objects capable of DNA binding for prolonged periods. Remove colocalized supercoils. | Kim. Data relating initiation and elongation rates [11]. |
| Sevier [13] | 2022 | Instant. | 2 steps. Reversible binding, SC-dependent DNA melting. | Slows with sigmoidal dependence on torsional stress. | Yes, according to a physical model of RNAP drag. | Yes; decay exponentially. | Stochastically relaxes DNA by some factor. | None. |
| Boulas [14] | 2023 | Instant. | 3 stages. Stage 2 has SC cutoff. | Constant rate. Stalls under sufficient upstream or downstream torque. | No. | No. | Consider specific and nonspecific activities of TopoI and gyrase. Instantaneous binding/unbinding. | Performed original experiments on plasmid containing topologically isolated gene. |

**References**

1. Sevier SA, Levine H. (2018) Properties of gene expression and chromatin structure with mechanically regulated elongation. Nucleic Acids Res. 6: 5924–5934. 10.1093/nar/gky382.

2. Marko JF. (2007) Torque and dynamics of linking number relaxation in stretched supercoiled DNA. Phys Rev E Stat Nonlin Soft Matter Phys: 021926. 10.1103/PhysRevE.76.021926.

3. El Houdaigui B, Forquet R, Hindré T, Schneider D, Nasser W, et al. (2019) Bacterial genome architecture shapes global transcriptional regulation by DNA supercoiling. Nucleic Acids Res 47: 5648–5657. 10.1093/nar/gkz300.

4. Chong S, Chen C, Ge H, Xie XS. (2014) Mechanism of transcriptional bursting in bacteria. Cell 158: 314–326. 10.1016/j.cell.2014.05.038.

5. Ahmed W, Sala C, Hegde SR, Jha RK, Cole ST, et al. (2017) Transcription facilitated genome-wide recruitment of topoisomerase I and DNA gyrase. PLoS Genet 13: e1006754. 10.1371/journal.pgen.1006754.

6. Crozat E, Philippe N, Lenski RE, Geiselmann J, Schneider D. (2005) Long-term experimental evolution in escherichia coli. XII. DNA topology as a key target of selection. Genetics 169: 523–532. 10.1534/genetics.104.035717.

7. Lenski RE, Rose MR, Simpson SC, Tadler SC. (1991) Long-term experimental evolution in escherichia coli.i. adaptation and divergence during 2,000 generations. Am Nat 138: 1315–1341.

8. Barrick JE, Yu DS, Yoon SH, Jeong H, Oh TK, et al. (2009) Genome evolution and adaptation in a long-term experiment with escherichia coli. Nature 461: 1243–1247. 10.1038/nature08480.

9. Ancona M, Bentivoglio A, Brackley CA, Gonnella G, Marenduzzo D. (2019) Transcriptional bursts in a nonequilibrium model for gene regulation by supercoiling. Biophys J 117: 369–376. 10.1016/j.bpj.2019.04.023.

10. Tripathi S, Brahmachari S, Onuchic JN, Levine H. (2022) DNA supercoiling-mediated collective behavior of co-transcribing RNA polymerases. Nucleic Acids Research 50: 1373–1388. 10.1016/j.celrep.2022.111492.

11. Kim S, Beltran B, Irnov I, Jacobs-Wagner C. (2019) Long-distance cooperative and antagonistic RNA polymerase dynamics via DNA supercoiling. Cell 179: 106–119.e16. 10.1016/j.cell.2019.08.033.

12. Geng Y, Bohrer CH, Yehya N, Hendrix H, Shachaf L, et al. (2022) A spatially resolved stochastic model reveals the role of supercoiling in transcription regulation. PLoS Comput Biol 18: e1009788. 10.1371/journal.pcbi.1009788.

13. Sevier SA, Hormoz S. (2022) Collective polymerase dynamics emerge from DNA supercoiling during transcription. Biophys J 121: 4153–4165. 10.1016/j.bpj.2022.09.026.

14. Boulas I, Bruno L, Rimsky S, Espeli O, Junier I, et al. (2023) Assessing in vivo the impact of gene context on transcription through DNA supercoiling. Nucleic Acids Res 51: 9509–9521. 10.1093/nar/gkad688.
