# Supplementary material for: Differential effect of supercoiling on bacterial transcription in topological domains
Source: PLoS Comput Biol. 2025 Nov 11;21(11):e1012764. doi: 10.1371/journal.pcbi.1012764 (PMC12622846; doi:10.1371/journal.pcbi.1012764)
Supplement: S2 Table — (DOCX) [file pcbi.1012764.s002.docx]

**S2 Table. Sources and Conditions of Expression Data.**

| **Organism** | **Study** | **Drug** | **Dosage** | **Duration** | **Growth phase** | **Total number of genes with expression values** | **Number of DE genes** | **Correlation coefficient** | **P-value** |
| --- | --- | --- | --- | --- | --- | --- | --- | --- | --- |
| *E. coli* | Blot et al. 2006 [1]. | Norfloxacin | 20 ug/ml | 5 min | Exponential | 4314 | 634 | -0.113 | 4.433e-03 |
|  | Peter et al. 2004 [2]. | Novobiocin | 100 ug/ml | 5 min | Exponential |  | 266 | -0.332 | 3.010e-08 |
| *S. enterica* | Gogoleva et al. 2020 [3]. | Novobiocin | 500 ug/ml | 60 min | Exponential | 4565 | 3296 | -0.175 | 5.769e-24 |
| *D. dadantii* | El Houdaigui et al. 2019 [4]. | Novobiocin | 100 ug/ml | 15 min | Exponential | 4351 | 1181 | -0.141 | 1.173e-06 |
|  |  | Novobiocin | 100 ug/ml | 15 min | Stationary |  | 1620 | 0.019 | 4.537e-01 |
|  | Pineau et al. 2022 [5]. | Seconeolitsine | 50 uM | 15 min | Exponential |  | 575 | -0.412 | 5.092e-25 |
|  |  | Seconeolitsine | 50 uM | 15 min | Stationary |  | 329 | -0.410 | 8.665e-15 |
| *Streptococcus pneumoniae* | De la Campa E-MTAB-141 [6]. | Novobiocin | 40 ug/ml | 15 min | Exponential | 1888 | 1293 | -0.261 | 1.177e-21 |
|  | De la Campa GSE77748 [7]. | Seconeolitsine | 8 uM | 15 min | Exponential |  | 1359 | -0.094 | 5.504e-04 |
| *Mycoplasma pneumoniae* | Junier et al. 2016 [8]. | Novobiocin | 50 ug/ml | 30 min | Exponential | 1034 | 313 | -0.614 | 1.809e-32 |
| *Synecochoccus Elongatus* | Vijayan et al, 2009 [9]. | No | Correlation with circadian cycle | - | - | 2529 | 2527 | -0.088 | 9.848e-06 |

**References**

1. Blot N, Mavathur R, Geertz M, Travers A, Muskhelishvili G. (2006) Homeostatic regulation of supercoiling sensitivity coordinates transcription of the bacterial genome. EMBO Rep 7: 710–715. 10.1038/sj.embor.7400729.

2. Peter BJ, Arsuaga J, Breier AM, Khodursky AB, Brown PO, et al. (2004) Genomic transcriptional response to loss of chromosomal supercoiling in escherichia coli. Genome Biology 5: R87.1–R87.16. 10.1186/gb-2004-5-11-r87.

3. Gogoleva N, Kravchenko U, Nikolaichik Y, Gogolev Y. (2020) Transcriptomic dataset of wild type and phoP mutant pectobacterium versatile. Data Brief 32: 106123. 10.1016/j.dib.2020.106123.

4. El Houdaigui B, Forquet R, Hindré T, Schneider D, Nasser W, et al. (2019) Bacterial genome architecture shapes global transcriptional regulation by DNA supercoiling. Nucleic Acids Res 47: 5648–5657. 10.1093/nar/gkz300.

5. Pineau M, Martis B. S, Forquet R, Baude J, Villard C, et al. (2022) What is a supercoiling-sensitive gene? insights from topoisomerase I inhibition in the gram-negative bacterium dickeya dadantii. Nucleic Acids Res 50: 9149–9161. 10.1093/nar/gkac679.

6. Ferrandiz M, Martin-Galiano AJ, Schvartzman JB, de la Campa AG. (2010) The genome of streptococcus pneumoniae is organized in topology-reacting gene clusters. Nucleic Acids Res 38: 3570–3581. 10.1093/nar/gkq106.

7. Martin-Galiano AJ, Ferrandiz MJ, de la Campa AG. (2017) Bridging chromosomal architecture and pathophysiology of streptococcus pneumoniae. Genome Biol Evol 9: 350–361. 10.1093/gbe/evw299.

8. Junier I, Unal EB, Yus E, Llorens-Rico V, Serrano L. (2016) Insights into the mechanisms of basal coordination of transcription using a genome-reduced bacterium. Cell Syst 2: 391–401. 10.1016/j.cels.2016.04.015.

9. Vijayan V, Zuzow R, O'Shea EK. (2009) Oscillations in supercoiling drive circadian gene expression in cyanobacteria. Proc Natl Acad Sci USA 106: 22564–22568. 10.1073/pnas.0912673106.
