# Supplementary material for: Differential effect of supercoiling on bacterial transcription in topological domains
Source: PLoS Comput Biol. 2025 Nov 11;21(11):e1012764. doi: 10.1371/journal.pcbi.1012764 (PMC12622846; doi:10.1371/journal.pcbi.1012764)
Supplement: Attachment — Submitted filename: 2025-08-06_single-gene-rebuttal.pdf [file pcbi.1012764.s012.pdf]

Dear editor and reviewers,

We appreciate your additional feedback on our manuscript. Please find our responses below.

Reviewer's Responses to Questions

Reviewer #1: The authors have adequately addressed all my concerns from the previous review cycle. I thank the authors for their responsiveness and willingness to consider my points.

I just seek clarification on one aspect. I am not sure what is meant by a deterministic-stochastic model. Is this a Gillespie simulation in which all the deterministic processes are stochastic processes with probability mass function of 1.0 (almost sure occurrence)? I found the discussion on lines 315-317 a bit vague still on this point. Also, the choice of the rate functions for the stochastic processes, specifically gyrase and topoisomerase binding seemed unclear.

We decided not to refer to our model as a Gillespie simulation because there are many differences between our system and a pure Gillespie algorithm, even though our model could be interpreted as a tau-stepping Gillespie algorithm that introduces several deterministic processes, which are evaluated at the end of each time step. We decided to term our model as “deterministic-stochastic” because it considers some processes stochastically (e.g., initiation, topoisomerase activities), while others deterministically (e.g., elongation, stalling).

Specifically, our model considers the following steps in order:

(1) Initiation
(2) Topoisomerase activities
(3) Elongation
(4) Termination

Initiation and Topoisomerase activities are simulated stochastically, randomly occurring in a fraction of timesteps. Elongation and termination occur deterministically, depending on defined criteria (an acceptable supercoiling SC level and an RNAP molecule located at the terminator, respectively). Deterministic processes are simulated using separate methods from the stochastic elements and do not utilize any probability mass function. We have revised lines 323-328 (copied below in italics) to emphasize that stochastic events occur before deterministic events within each timestep. Additionally, we have added a caption to Table 1 to explain each of the variables considered within our rate functions.

Initiation, topoisomerase activities, and RNA decay are simulated stochastically (Table 1) by randomly selecting the waiting time before each upcoming event from the appropriate time distribution. Within each 0.04 s timestep (the time for an RNAP molecule to elongate by one bp), we resolve any stochastic events scheduled to occur before simulating elongation and termination deterministically. Supercoiling levels are updated instantaneously whenever any SC-altering event occurs.

In Figure 6C-6D the authors describe simulating multiple genes using the deterministic-stochastic model to see how topoisomerase inhibitors affect transcription levels. I am unclear as to how the authors accounted for the binding of transcription factors and other heterogeneous effects in simulating each gene. Is this a generic "one gene" fits all model? Or is there something about each gene simulated that makes it actually represent a given gene in each of the bacterial species simulated across their transcriptome? This point seems critical to clarify.

This point is excellent. We did not consider the binding of transcription factors or simulate individual genes for each organism, as we intended to evaluate the role and contribution of supercoiling in creating regulatory effects. In other words, we are interested in finding out whether this simple model, a “generic”, one-gene model without considering transcription factor binding and other complex regulatory elements in cells, could recapitulate experimental measurements qualitatively. We showed that the model could indeed predicate similar trends for the responses of strong, intermediate, and weak genes to novobiocin and seconeolitsine, despite that the experimental data was the result of many other confounding factors. This result suggests that supercoils have a strong effect in cells and that our model can largely explain experimental observations. In the revised text, we now more clearly state the simulations we ran and the statistics we calculated from those simulations (lines 726-767, copied below in italics).

We note that while our model does not consider transcription factor binding and other complex regulatory elements in cells, it is meaningful to compare the simulation and experimental results qualitatively to identify the role and contribution of supercoiling in gene regulation. Furthermore, since the exact effect of drugs on topoisomerase activity in vivo is not precisely known (and likely variable in our dataset), we can only derive a qualitative comparison. We ran simulations mimicking an (arbitrary) 5-fold reduction in either enzyme, considering the log2 fold change in expression level induced in three representative genes of strong, intermediate, and weak promoter strengths (S6 Fig.). Since we are interested in the relative effects of topoisomerase inhibition on genes of varied strengths, we calculated the ratio of this log2 fold changes between strong and weak genes, shown in Fig. 6C-D (comparable to Fig. 6B) for simulations across all gyrase and topoisomerase activity values.

After a minor revision addressing these points, I would be happy to review one last time for final publication.

Thank you for your constructive suggestions. We hope this revision has added clarity and addressed your concerns adequately.

Reviewer #2: The authors have substantially improved the manuscript and provided improved clarity of the figures. While there are still opportunities to improve the message of the figures, the accompanying text is sufficient for the reader to gain the main ideas. The message of figure 4 is particularly improved and the idea of changes in length and topo activity influencing one another is clear. The Fano number is an appropriate metric for these simulations. CV is nice but Fano is proabably better and more appropriate. The work is fine currently fine as is.

We particularly enjoyed this line from the authors as often the appreciate between plasmid and chromosomes is not fully appreciated. "Within the current framework, the comparison suggests that topoisomerases affect transcription in a different manner in the plasmid employed in the latter study compared to most chromosomal genes analyzed in the transcriptomics data" Thanks for highlighting!

Below are minor suggestions for a bit more visual clarity.
- 1D could use some labels / sizing. The labels \sigma_s -\sigma_s are tiny for the black dashed lines.

We agree. We have implemented these changes to make the figure labels easier to read.

- 1E could use some highlighting –

We now interpret Figure 1E in the context of elongation stalling within the corresponding figure caption. The RNA and RNAP copy numbers are plotted over time primarily to visually capture transcriptional bursting, as discussed in the text corresponding to Figure 5.

-Figure 2 Label the red line as -\sigma_s –

Done.

-Line 483: 3D? You mean Aiv? - Figure 4: scaling seems to be off.

Thank you for spotting this typo, which we have corrected. However, the scaling of Figure 4 appears correct on our computers, so we are unsure what issue the reviewer is referring to.

-Fig 6A: needs an updated y-axis label "fold change _upon gyrase inhibition_"

We agree that this would make the figure clearer and have updated the y-axis label.

- Figure 6: it would be nice to rearrange the overall figure, to have one row for all of the gyrase inhibition + the modeling result, then another row for all of the topisomerase inhibition + the modeling result.

We have rearranged this figure and updated the figure caption to match these changes.

-Line 449??? Somehow an emoji snuck in

We apologize for this incorrect symbol that was introduced when converting between our working .docx file and the .pdf file you viewed. This symbol has been corrected (now on line 470).

Reviewer #3: The authors have provided a major revision that addresses all my comments and criticisms. I find the resulting manuscript clearer, more accurate, less emphatic, and more nuanced regarding the level of predictions this type of model can offer.

Overall, the study presents convincing new results, showing that models based on the

physics of RNAP stalling—and how it is handled inside the cell—are becoming increasingly promising for understanding many aspects of gene expression. For this reason, I believe this work deserves publication.

More personally, and in response to the authors' replies, I still feel that stating all models are "broadly" equivalent does not do justice to modeling efforts and tends to downplay the importance of model assumptions and their predictive power.

We agree that, in our attempts to convey the similarities between these models, we brushed over the differences and may have implied a false equivalency. We have reduced these remarks and just conserved one sentence (at the beginning of Methods) evoking a conceptual similarity while mentioning differences (l. 153-154).

Minor comments:
- Lines 168-169: English issue

We have split this sentence to increase clarity (lines 168-171).

- Line 256: "Supercoiling" should be lowercase.

We have corrected this typo.

- Lines 261-267: This discussion directly concerns the code on which the authors have based their work (i.e., our model [21] based on RNAP stalling). As explained in [22], and as I have personally discussed with Sam Meyer, this code/model assumes that upstream and downstream torques act independently. This assumption differs from other models based on RNAP stalling. The impact of this choice on the results has never been studied and should be addressed in future work. Therefore, I find it somewhat awkward/misleading to refer to this aspect by stating that "some models have adopted a similar approach to model the stalling of elongating RNAP molecules [21]".

We have revised this section (line 267-270) to avoid framing [21] as an entirely separate model. We have also explained that the treatment of upstream/downstream torques independently is advantageous, as it allows us (as in [21]) to use values taken directly from experiment, and we additionally note that future research should consider the consequences of this decision.

- Line 449: Wrong symbol.

We corrected this wrong symbol – see our response to review #2 above as well.
