# Supplementary material for: Differential effect of supercoiling on bacterial transcription in topological domains
Source: PLoS Comput Biol. 2025 Nov 11;21(11):e1012764. doi: 10.1371/journal.pcbi.1012764 (PMC12622846; doi:10.1371/journal.pcbi.1012764)
Supplement: Attachment — Submitted filename: 2025-10-25_rebuttal-letter.pdf [file pcbi.1012764.s013.pdf]

Dear editor,
We thank you and the reviewer for the attention given to improve the manuscript. Please find our answer to all requests.

Reviewer #1: This constitutes a final, editorial review for Boaz, Nicolas, Jie, and Sam. I find the paper to be very compelling and the results to be extremely useful for thinking about transcription, DNA supercoiling, and topoisomerase-based regulation. The paper presents a mechanism to explain and control the size of transcriptional bursts and a means to capture the dual-side tapered effect of optimal supercoiling on transcription initiation and elongation, as well as topoisomerase activity. The paper presents a novel mathematical model, based on a combination of deterministic and stochastic reactions. I recommend this paper for eventual publication, but only after a major revision to address extensive editorial issues (grammar, typos, and expository gaps).

*In the abstract, update this sentence to express the following: "SC therefore regulates transcription in the absence of transcription factor binding, in the context of chromosomal topological domains and the actions of topoisomerases."

We reworked the abstract, considering the comments below.

Too often, overly strong claims or sweeping generalizations are asserted that need to be softened to reflect prior work or prior findings.

*Line 48: Clarify domain size of what?

The topological domain containing a gene; we have rephrased this sentence for clarity.

we developed a quantitative model to describe SC-coupled transcription." (This is not novel, see Galloway, Yeung for two recent examples and there are older examples in the literature). The authors do a good job of identifying some gaps in the literature but then do not connect those gaps with what they say they will accomplish in the paper. Differentiate the work from what has been done.

Yes, several prior models of SC-coupled transcription exist. We do not claim that us having developed such a model is novel; rather, the experimental phenomena we are able to reproduce and the mechanisms we propose for these data are the novel contribution of our work. We emphasize this contribution more heavily now in the abstract.

*Line 51: Given the comments from the authors in response to my previous review, this sentence in the abstract should be "transcription of a single isolated gene with varying promoter strengths and in a topological domain of varying sizes" should say something to the effect of a "we formulate a generalized, single gene model, agnostic to the specific regulatory characteristics of various gene networks within single prokaryotic and eukaryotic cells, to examine the predictive power of a model that articulates the effects of DNA supercoiling,

enzymatic topoisomerase activity, and “domain size” and find that the model is surprisingly accurate in predicting XYZ ….”

We agree that this sentence should be rephrased. However, we do not claim that our model is agnostic to the difference between prokaryotic and eukaryotic cells, as our work is focused on bacteria only. We do, however, clarify that our model is independent of the effects of transcription factor binding, as this helps get across the importance of SC. We have largely rewritten the abstract to emphasize the specific novelty of our work.

*Line 56: Several bacterial species should be rephrased with a statement about the diversity of phyla or taxonomic classification of the species - e.g., we examined both gram-positive and gram-negative, or we examined a range of soil borne, water-borne, and mammalian host associated microbes…
We have specified “evolutionarily distant species”, since they span a large part of the bacterial phylogenetic tree. More precision is given in the Results about the specificities of each specie.

*What is meant by “a relevant parameter range”?

We have rephrased this part. We replicate the observed behavior only within a part of the parameter space, which gives an indication on the biologically relevant values of the parameters.

*What is the global relationship? Just state the type of relationship in the abstract.

We now specify that we are discussing “the repression of highly expressed genes”.

*A period is missing after the first sentence of the paper.

We apologize for this mistake introduced in typesetting our in-text references.

*A period is missing after the second sentence of the paper.

See above.

*Line 85: S-induced torque can stall what?

The elongation of RNAP.

*Line 102: Comma splice (grammatically incorrect comma)

The comma has been removed.

*Line 111: [57] also develops a mathematical framework for modeling bacterial transcription and SC, but it does not take a computational biophysics focus.

We have added this reference (now [27]) to this list of models (line 116).

*Line 113: [57] explicitly modeled the effect of supercoiling on transcription in two stages, initiation and elongation with separate regulatory dynamics. The two stages were modeled as deterministic though, which is probably inaccurate and too coarse grained at the molecular level.

We have added this model to the list of models that consider the regulatory effect of SC on both initiation and elongation (line 118).

*Line 114: Enzymatic terms of gyrase and TopoI activity were included in [57] but they did not take into account the bell-curve response of catalytic enzymes around optimal supercoiling setpoints.

We now add this reference to the models which consider gyrase and TopoI activity (lines 119).

*Line 117: [57] explicitly shows comparisons of quantitative fits of experimental measurement data with predictions for a transcription-supercoiling model. The model is able to predict the response in cell-free systems quite well, but it was not able to account for the heterogeneity in response when considering transcription factors. The model also made structured assumptions about gyrase and topoisomerase's response curves, which were hypothetical, at best, since the authors lacked gyrase and topoisomerase response curves for their plasmid studies.

We now refer to [57] (now [27]) here (line 123).

*Line 126-127: Again, reference [57] is completely omitted when the entire point of the study was to understand how neighboring genes impacted each other in various orientations on plasmids. Additionally, [57] shows how neighboring gene orientation can be used to dramatically improve performance of transcription in the toggle switch circuit.

We have added the reference (line 132).

*Line 128: "To the best of our knowledge"

We now use this phrasing (line 134).

*Line 134: The introduction to the single gene approach its quite abrupt. I think there is a lot of context missing here as to what one means by a "single-gene approach". There should be some brief discussion about how this is meant as an abstraction of most or all genes in the genome, as conveyed by the authors in the helpful replies to this reviewer. As is, the phrase is referenced as if it is a common technique/modeling approach in the field and I doubt that this is the case.

Perhaps one could invoke the “world is resistor” analogy that electrical engineers use, where a dramatic simplification is applied to the excluded elements of the model, with a primary focus on one component, the gene of interest.

We agree that this expression was very unclear. We now explain our approach, starting from the previous works focusing on 2 or 3 genes and exhibiting a remarkably complexity, which led us to focus on a simpler system to reduce the number of parameters and quantitatively explore the parameter space (line 139-147).

*The introduction of the modeling is still extremely vague. The authors should clearly state upfront what the model is, what type of model class it is, express the model equations for the deterministic components and the reaction kinetics for the stochastic components. Instead, the authors introduce the model by drawing comparisons to references in the literature, suggesting what it adds or what it is not. This is a terribly confusing way to introduce a mathematical model. For a sound example of how to introduce a mathematical model, refer to this paper by Sam Meyer and Chaoming Song (2010) or this paper by Sam Meyer and Jost (2010).

We have entirely rewritten the introduction of the model to address this comment.
The Results section now starts with a high-level presentation of the model that refers to the Methods for details: (lines 434-444).

The Methods section starts with a general but more technical presentation of the model (lines 167-177), followed by paragraphs describing elements in more detail, and the comparison to previous models was moved to a dedicated paragraph in addition to S1 Table.

*Line 172-Line 183: This reads more like an appropriate introduction of the model but it still lacks detail and digresses into a discussion of literature and potential variations on the model and justifications for the current choice (where the reader is still confused as to what the current choice actually entails). We also get a stronger sense of the model content in the caption of Figure 1, but this exposition is not had in the main text of the paper. It should be in both.

See above. We have reorganized the Methods and Results sections to address the reviewer’s legitimate concern.

*Line 254: What is the justification for using beta_i = 0.005? It doesn’t necessarily follow from what is stated in Line 253. Please be more careful and explicit in your logical reasoning.

This value was chosen because it aligned closely with the data from [37], mentioned in the previous sentence. We changed our phrasing to make this clearer (line 248-249).

*Line 257: The postulated 25 bp elongation rate could use a literature reference.

We copied the reference provided in Table 2 here (line 268).

*Line 286 should have commentary about what you are not using the coefficient of variation, a scale-free metric for intrinsic, normalized variation of a random process/variable.

The natural choice is the Fano factor, which is one for any Poisson process, regardless of the mean. Therefore, the Fano factor lets us easily separate Poisson vs. Bursty transcription. We now state this in our manuscript. (lines 284-285).

*Line 322 should state any potential package dependencies and provide potential links to python code. Why was the simulation split across two computing languages?

We now specify that we used NumPy 2.1.3 (line 319). The simulation was completed in python, while analysis was completed in R; this was a matter of convenience and does not affect the results.

*What is the justification for what is simulated deterministically versus stochastically? This seems somewhat arbitrary since elongation and termination could be viewed as stochastic processes comparable to initiation and topoisomerase binding(which were simulated stochastically). Why is RNA decay simulated stochastically while SC diffusion is deterministic? Both seem like first order processes — what is the deciding criterion?

SC diffusion is treated as instantaneous (at the timescale of transcription and over our domain lengths).
As for our other modeling choices, we have to make a tradeoff between model complexity and accuracy. We treat RNA decay as a stochastic process because this directly increases the noise of the RNA copy numbers, relevant to our analysis, and adds very little model complexity based on parameters we have experimental measurements for (the RNA lifespan).
Termination: we focus on strong promoters which are approximated as deterministic, since we are not interested in transcriptional readthrough in this study.
Elongation could be modeled as a stochastic process, but that would require adding additional, experimentally unknown parameters. We therefore choose to make simplifying assumptions. In our methods section, we now discuss our decision to consider instantaneous SC diffusion (lines 175-177) and deterministic elongation (lines 266-271).

Line 640 etc: Define what scenario 1 and scenario 2 and scenario 3 actually entail rather than just referring to their existence and the corresponding outcomes.

We agree that we should have made what these scenarios represent clearer in the text; we have added descriptions (lines 663 – 668).

*Line 785: The model is not fully stochastic. Please update to be accurate and consistent with the definition in the paper. It is a hybrid deterministic-stochastic model.

Upon reconsideration, we decided that using a more general term "computational model" would be more appropriate at this specific location (line 816). The distinction between deterministic and stochastic events is presented in the Methods section.

*Line 870: What is this line trying to say? This should be the final draft of the paper and it seems like the writing is still in rough draft form.

We removed this repeated sentence. We have also generally proofread the full manuscript to avoid similar mechanical issues introduced during the editing process.

*Does the paper need a conclusion section?
.
The submission guidelines for PLoS computational biology do not include a conclusion section in the "manuscript organization" guidelines (https://journals.plos.org/ploscompbiol/s/submission-guidelines#loc-manuscript-organization), so we choose not to include a conclusion section. We have, however, carefully revised our abstract to make our conclusions clearer.

*Supplemental Figure 1: Should there be error bars?

While error bars may be useful, this figure relies on data from prior studies, for which error bars are unfortunately not available, but we do not expect them to affect the main conclusions we are trying to replicate.

*Supplemental Figure 2: What does the red time-series trace on the "Weak Promoter" simulation end prematurely?
This could be potentially annotated or overlayed to explain the significance of the premature end of the red trace.
The red trace does not end; it simply overlaps with the blue trace. We have now specified this in the figure caption, where we now interpret this as the period in which no RNAPs are bound to the gene.

All in all, I feel this paper should be published in PLOS Comp Bio after a major revision. I encourage the lead author to refine the quality and polish of the writing in this paper, to abide the highest of academic standards. I would be happy to review the paper one last time, if needed. Thank you for reading.

Reviewer #2: Congrats on the interesting work!
Reviewer #3: The new manuscript is ready for publication.
