# Supplementary material for: Differential effect of supercoiling on bacterial transcription in topological domains
Source: PLoS Comput Biol. 2025 Nov 11;21(11):e1012764. doi: 10.1371/journal.pcbi.1012764 (PMC12622846; doi:10.1371/journal.pcbi.1012764)
Supplement: Attachment — Submitted filename: Rebuttal Letter.docx [file pcbi.1012764.s011.docx]

Dear editor and reviewers,

We thank you for your feedback on our manuscript. Please find all answers below.

5)  Thank you for stating that "All data files are available from the Zenodo database at DOI: 10.5281/zenodo.14586107." This link reaches a DOI Not Found page. Please amend this to a working link or provide further details to locate the data.

Thank you for pointing that mistake. We corrected the link: “https://doi.org/10.5281/zenodo.14586118“.

Reviewers' comments:

Reviewer's Responses to Questions

Reviewer #1: The authors present a stochastic, Gillespie simulation based approach for simulating the differential effects of positive and negative supercoiling on bacterial transcription in confined topological domains. The simulation results are intriguing and allow a computational framework to explore how fluctuations in gyrase and topoisomerase concentrations cause polymerase stalling. The paper is well written, well motivated and deserves consideration for publication. Overall, I recommend one cycle of revision and re-review.

We thank the reviewer for the positive feedback.

My primary critique of the paper, which is mostly minor, is that the modeling framework used in this paper is referred to as the Gillespie algorithm (or the SSA), but none of the conventions for presenting a Gillespie stochastic simulation are followed. It is standard to draw the chemical reaction network as a figure, to list the corresponding chemical reactions, and then to write down a table of the propensity functions associated with each reaction channel.
Simulation results and parametric tables are usually plotted (this part is done correctly in the paper). None of these conventions are observed which makes it very difficult for a reviewer to evaluate technical correctness, short of a full exploration and reverse engineering of the code. The notation in the code needs to reflect the notation used in the paper (which is basically absent since no presentation of the equations or reactions are made), but this is a minor omission that can be easily fixed.

Thank you for this comment. Our simulation does not utilize a full Gillespie algorithm, but instead a combination of stochastic and deterministic elements. We borrowed some methods from the Gillespie algorithm for computational efficiency when considering the timing of stochastic events. For example, our implementation attempts initiation at time intervals drawn from an exponential distribution, with the success rate depending on the local supercoil (SC) level. The same applies to topoisomerase activities. Therefore, we did not include a chemical reaction network as we did not track our simulation state with the state vector typical of a Gillespie algorithm. In the revision, we clarified this issue in lines 315-317 and also added a new Table 1, which lists and explains the deterministic and stochastic elements of our model and describes the rate at which these events occur.

Qualitatively, the results are very interesting. I especially find the stalling characteristic of the simulation framework interesting. It is worth noting that there are earlier works modeling the supercoiling-dependent nature of transcription initiation and elongation, especially as they related to gene expression in cell-free and in vivo bacteria (see the work of the Murray group by Yeung et al).

We added the citation to 10.1016/j.cels.2017.06.001, which we had curiously missed in our list of references even though it had been a source of inspiration for this work. It indeed shows that the phenomenon occurs both in and outside the cell, and in absence and presence of translation. We cite this paper in our discussion of multiple neighboring genes (Discussion, lines 843, 851-854).

I was a bit confused as to why the authors used the Fano factor (a measure of deviation from a Poisson distribution) rather than the coefficient of variation to measure noisiness. It's clear the authors were trying to draw a connection with the literature, but it seems like the COV would be a scale-free metric for the amount of noise or burstiness in transcription.

We had indeed included both the Fano factor (Fig. 5) and the noise (Figure S4) in the initial manuscript. We agree that the Fano factor is not an obvious measure for noisiness, but we intended to use it to compare the deviations from Poissonian distributions for all promoters. Since it is useful to keep both measures, we now moved the noise figure to a new Fig 5A, justifying the use of the Fano factor in Fig 5B.

Lastly, it seems the top-down ('omics") approach to validating what is a reductionist model of single-gene transcription seems a bit mismatched. This part of the paper seemed underdeveloped, so I would suggest that the authors exclude the section all together or expand on that section so that the reasoning is much more clear. Further, the number of parameters in the model seem far more than what the data can constrain, so it seems like it would be hard to justify the observations in nature as uniquely explained by the predictions from the model. Other parameterizations of the same model might yield distinct, contradictory predictions - this does not show the model is explanatory nor non-explanatory since we don't have a great sense of the perfect parameterization of the model. The core issue is model identifiability with such a generic collection of datasets from the literature. I don't have a solution for this challenge and while I find the comparison interesting, this may be a point of scrutiny/criticism from the scientific public.

We thank the reviewer for this excellent point. We indeed have discussed among ourselves multiple times and agree that this section is insufficient. Our final consideration is that even if the comparison with *in vivo* data has limits due to the many unknown features affecting those data, it is still useful and may inspire future studies with a more systematic approach.

To mitigate the concerns and improve the comparison, in the revision we completely rewrote this section’s result and a significant portion of the corresponding discussions based on the following new additions. First, we computed the relative expression log2-ratios between weak and strong genes. The ratio can be directly compared between simulation and transcriptomics data. Second, we added a comparison to the data referred to by reviewer 3, from a plasmid-borne promoter in a topological domain of various sizes. Third, we now described more precisely what range of the parameter space reproduces the observed behavior. Finally, within our framework, we verified that the results were robust when changing the distance to domain barrier, which is an influential parameter. We hope these new additions now clearly state the capacity and limits of the model.

Reviewer #2: Goldberg et al. set out to model DNA supercoiling and examine the effects of two types of topoisomerases: topoisomerase I and gyrase. They specifically modeled the recruitment of these enzymes to supercoiling sites as a function of supercoiling density and sign, resulting in complex, non-monotonic dynamics. The focus of their study was on the activity of a single gene, where they varied the strength of the promoter and the size of the domain.

The authors demonstrated that there is an optimal level of supercoiling—specifically negative supercoiling—for maximizing transcription rates. They found that weak promoters are not affected by slow elongation speeds but are limited by initiation rates. In contrast, strong promoters are constrained by RNA polymerase stalling. These differences lead to distinct sensitivities to topoisomerase I and gyrase for various promoters. The data suggest that there is an optimal level of activity for topoisomerase I and gyrase to achieve the highest level of gene activity, which may vary depending on the initiation rates of the promoters, essentially defining their strength.

Regarding domain size, smaller regions result in fewer relaxation events and more stalling. As a result, strong promoters in smaller domains are limited by topoisomerase activity. Conversely, larger domains favor transcription with lower topoisomerase I levels and higher gyrase levels. Interestingly, the authors show that strong promoters exhibit low noise (Fano factor below 1), indicating more coordinated bursting due to synchronization of RNA polymerase binding times. Finally, the authors examined multiple bacterial species to identify trends in expression patterns upon topoisomerase and gyrase inhibition, highlighting differences between strong and weak promoters.

We thank the reviewer for the excellent and succinct summary of our work.

Main points

1. There should be schematics to explain the models for the main figures. Figure four could especially benefit from cartoons illustrating what each row is trying to represent with respect to changes in the model. This will help with interpretation of the data. Similarly figure 3 could benefit from some cartoon graphics helping the reader to appreciate what is happening in the panels of data. Potentially both what is modeled and what is observed. Figure two could have a cartoon above the left panel of what the input distributions are schematically (e.g. delta function spikes at specific topoisomers, or a normal distribution).

Thank you for this excellent suggestion. We have now updated the three figures to improve readability. In Figure 2, we included plots of delta functions and normal distributions to indicate considered topoisomerase activities. In Figure 3, we added equations showing the calculations we used (also in Figure S4). Finally, in Figure 4, we introduced cartoon descriptions of the described barrier distance and topoisomerase activity changes.

2. Heatmaps are not the most effective way to present data. The text for the results convincingly supports its arguments, but Figures 3 and 4 obscure these arguments. For example, the authors argue in Figure 3 that there is an optimal amount of Top1 activity for a given promoter strength. This is visible in the heatmaps, but would be clearer if, for a few choices of gyrase activity, plot the transcription rate (or elongation speed, etc) as line charts. Effectively, take a cross-section of the heatmaps in order to clearly show non-monotonic behavior.

Another possibility is to make graphs that look like the data presented in FIgure 6. A more natural way to understand the Figure 3 results would be a line plot with “promoter strength” on the x axis and “normalized transcription"/other variables on the y axis, with the use of different colors or linestyles to separate lines. All in all, being able to map a specific text claim to a specific, explicative plot would be great!

We thank the review for the excellent suggestions. We agree that the figures should better walk the reader through the main results. In the revision, we still show heatmaps but replace or complement most of them in Figures 3, 4, and S3 with line plots showing the results of varying TopoI or gyrase activity. We still include all original heatmaps in the supplemental information for reference. Accordingly, we revised the text to reflect the changes (results on “Regulatory effects of topoisomerases’ activities depend on promoter strength” and “Regulation by topological domain size”).

3. Do similar results in Figure 4 hold if the domain size is held constant at 10kb, but the topoisomerase rate is 0.1x or 10x? The authors argue that most of the domain size trends are because of the relative activity of the topoisomerases. Discussing this case would strengthen that argument.

We do in fact observe similar results to those in Figure 4 when we hold the domain size constant and vary the topoisomerase activities 10-fold in either direction. We show this in the new version of figure 4.

Relatedly, in Figure 4, why are the y-axis tick marks the same in rows B and D, in the cases with 10x and 0.1x topo activity?

The y-ticks are the same in all plots because we used the topoisomerase activities in the initial state to determine the axes. Since we altered the topoisomerase activities in the comparison, we need to choose whether the labels will show the initial or ending topoisomerase activities for the comparison, and we chose the former for consistency across panels.

3. The assumptions regarding polymerase drag should be explicitly stated. The assumption of constant linking number upon elongation (e.g. the only change in linking number constraints is a result of gyrase/Top1 activity) assumes that the polymerase is so drag-limited that only the DNA, not the polymerase, rotates. The authors recognize this assumption when they say “the frictional drag experienced by RNAP might be weaker due to the absence of translation and a less crowded environment compared to the model assumption.”, but it would greatly clarify the model development part if this assumption is made more explicit.

We agree that this important hypothesis was not emphasized enough. We now mention it and its implications for our simulation in the overview of the Methods section (l. 158-160), the Methods section on elongation (l. 252-254), as well as the Discussion (l. 847-848).

4. Plasmid modeled as circular? Highly distant boundaries are not likely to replicate the predictions of a circled plasmid outside of the single-transcriptional-unit case presented. Even in the single-gene case, stochastic motion of polymerases should cause a small but non-zero amount of supercoiling due to diffusion around the backbone. This likely does not affect the presented results, but the authors should potentially call out this limitation. The more common way to encode circular boundary conditions with a linear coordinate is to “reflect” the first and last polymerase linking number constraints past the edges of the domain as “ghost” linking number constraints.

We agree that the use of highly distant boundaries is a somewhat crude treatment. To examine the potential effect of the small but non-zero amount of supercoiling due to the diffusion around the backbone, we varied the (unrealistically large) distance to barriers and observed that the SC level changed by less than 0.0003 in these simulations, which will not induce stalling or meaningfully effect the initiation rate (l. 332-335). As we only used this situation for the special case of the *in vitro* transcription template, we consider the result equivalent to the reflecting boundary simulation. We now describe this treatment more explicitly in the methods section on “Transcription of a freely rotating plasmid without topoisomerases”.

5. 2. The authors should explicitly state that “we assume that the linking number of polymerases does not change upon elongation; it only changes upon gyrase/topo level. Because linking number is excess twist / distance, constant linking number upon elongation means that all of the rotation is going into supercoiling generation. In other words, this assumes that the polymerase is so drag limited that it does not rotate”. This explains the Figure 2 statement that “maybe certain regimes are not this drag limited”

Similar to what we described above to #3, we now put more emphasis on this hypothesis (lines 252-254) and discussed its relationship to Figure 2 (lines 434-436). We note that *“[the linking number of polymerases] only changes upon gyrase/topo level*” does not describe our system exactly, because the *Lk*_i_^(up)^ and *Lk*_i_^(down)^ of a polymerase can change if an adjacent RNAP molecule initiates or terminates. In this scenario, however, it represents a change to the length of a domain on which the linking number is being tracked rather than a change to the SC density on this domain.

Minor points
1. Check the references for number 51 line 777. I'm not sure this is the intended reference

The reference was in error and has been corrected.

2. In the results section for Figure 5, the authors should discuss that the Fano factor is not scale-free, e.g. to get meaningful Fano factors, you need to know the exact number of transcripts, which requires either simulations or some method that counts discrete number of transcripts. This could motivate the choice of experimental data that the authors used.

We now show both the normalized variance and the Fano factor in Fig. 5 (A and B, with the former being approximately inversely proportional to the mean expression) and mention that the Fano factor depends on the transcription strength for the Results section of figure 5 (l. 577-580) as well as the Discussion mentioning experimental measurements (l. 825-826).

3. In figure 5A/5C, potentially consider other, clearer plot types. Instead of an overplotted scatter plot in 5A, you could use faceted KDE plots to see how the overall distirbution shape changes. In 5C, overlapping barcharts obscure the marginal distribution changes. Consider a 1D KDE or a line-only histogram, so readers can clearly compare the Poisson distribution against the calculated distributions.

We considered alternatives to the overplotted scatter plot shown in figure 5A, such as 1D KDE plots. However, we found that due to a low quantity of data at high Fano Factors, either the KDE does not effectively communicate the data on either a linear or logarithmic x-axis (high Fano Factor points appears as isolated kernel functions). We have elected to keep this as a scatter plot to avoid confusion. For figure 5C, we decided that due to the discrete nature of our data and the Poisson distribution, a bar-histogram was the most accurate way to show the possible copy numbers.

Reviewer #3: Reviewed by Ivan Junier.

Several biophysical models have been proposed over the years to better understand how the topological constraints associated with transcription (and also replication) are managed in the cell and what their impact is on gene expression. The work of Goldberg et al. follows this line and aims to understand the implications of certain models that were recently developed by the authors (ref [23]) or by research groups like ours (ref [17]). In particular, these models have demonstrated their ability to quantitatively rationalize complex expression data where physical constraints are varied in the case of an isolated gene [17], which is exactly the experimental situation discussed in this work.

In this context, the proposed manuscript seems to me to be an interesting contribution to the understanding of these models. However, for this work to be publishable, important changes and additional analyses are required.

We thank the reviewer for the positive assessment.

First, the introduction of the paper is relatively vague regarding the range of models that have been proposed in the literature. A better contextualization is necessary. Otherwise, the authors’ model will appear as just another model. More specifically:
- A brief historical overview of the models seems necessary.

We agree that this part was insufficient. We have introduce a table summarizing previous models of transcription-SC coupling and their modeling decisions in order to better contextualize our research, as Table S1. In the main text, we summarize how our model relates to these previous works in Materials and Methods (l. 150-168).

- It is essential to explain the similarities and differences between the proposed model and the most recent ones [17] and [23]. With respect to [17], I see two major differences: i) the consideration of the processivity of gyrase activity, which effectively generalizes [17], and ii) the absence of specific activity for topoisomerases — upstream of the gene for TopoI and downstream for DNA gyrase, which thus reduces the generalization power compared to [17]. In [23], authors considered the additional possibility for the RNAP to rotate.

We now discuss these relationships between our work and these references, first briefly in the Introduction, then in the Methods section (l. 150-168), as well as in the new Table S1.

- The authors mention that one of the advances of their model is the consideration of the continuous response to SC of topoisomerase activity. However, in lines 167-169, the authors mention that their work is not sensitive to the discrete or continuous approximations of topoisomerase activities. I therefore conclude that there is no need to consider a continuous version that requires more parameters.

This is a good point, and we nuanced this sentence in the revised version. The value of the additional parameter (width of the sigmoidal curve) did not modify the overall results (within a reasonable range of values) so it does not really constitute an additional parameter, but it did improve the stability of the simulations by avoiding strong artificial changes in behavior induced by tiny changes in supercoiling values around the threshold values, and thus better represented the expected behavior of the real system. We stated that point more clearly (l. 163-168).

- The authors mention that they take into account the issue of gyrase topoisomerase binding rates (also in the abstract). However, their model does not separate the binding mechanism from the activity once bound. Therefore, the authors should explain this better, particularly that their model, like others before it, considers an effective approximation of these two process.

We agree that this process was not entirely clearly explained. Once bound, we allow gyrase to undergo a variable number of catalytic cycles, following experimental values of the distribution of this number, so we do separate binding and activity, which was absent in previous models. However, for simplicity, we simulate the whole round of catalytic cycles as instantaneous (together with binding), which is indeed an effective approximation, but differs significantly from previous models. In contrast, TopoI does only one cycle instantaneously once bound. We developed this description in the methods section on topoisomerases (l. 283-311).

Secondly, the authors justify the quantitative nature of their model by emphasizing its agreement with available experimental data. For instance, compared to Chong et al. [28], they also observe Fano factors around and above 2. However, I note that their distribution of transcript numbers is actually qualitatively different from that observed in [28]. In particular, as shown in Fig. 7B and Table S1 of [28], Chong et al. found a distribution of mRNAs with a very high number of cells showing no transcripts, which mathematically corresponds to a Poisson with zero spike distribution [28].

Thank you for pointing out this difference. We did not discuss it explicitly in the previous Discussion section. In the revision we now discuss that the Fano factors are in the right order of magnitude, but the number of mRNAs may differ – although these data do not provide a direct quantitative estimate of mRNA numbers (Discussion, l. 823-831).

More importantly, an important comparison is missing in the proposed manuscript: that with experimental results obtained in [17] where a systematic study of the effect of barrier distances (downstrean and upstream) is performed, i.e., just as the framework the authors consider. In particular, the authors should investigate the conditions to observe an absence of an effect of downstream length on the expression rate of an isolated gene (Figure 2A in [17]).

We agree that this comparison was missing in the initial manuscript. It is now shown in Fig. S7, which is referenced in the Results section when we discussed the relationship between our work and in vivo data. We did observe conditions in which only the upstream sensitivity is greater than one; specifically, for weak TopoI binding and moderate to strong gyrase binding.

Finally, the authors mention that their work “provides a unifying framework” (abstract and discussion). I do not agree. Indeed, in [17], it is shown that a quantitative explanation of the dependence on the size of the upstream domain requires considering the specific activity of TopoI, an operational mode that is not accounted for in the authors’ model.

We agree that the formulation was too strong, and nuanced it as requested by the reviewer. We also included the mentioned dataset that we had mistakenly not considered in the initial manuscript. It is true that the model does not quantitatively explain all considered datasets, so we removed that notion. It does unify a large set of observations from very diverse sources of data, which had not been attempted before. The modified text can be found in the Abstract (last sentence) and Discussion (l. 753-755).

The comparison to the study mentioned by the reviewer is now described in Fig. S7. The observed behavior is reproduced qualitatively, although not fully quantitatively. This outcome was expected since we built the model keeping the binding properties of topoisomerases as generic as possible, even though it is known that both gyrase and topoisomerase I can bind in a sequence-specific manner (in this case, as the authors of the original study did, introducing a binding term independent of barrier distance, but it may then depend on the particular sequences used *etc*). This point is now presented more precisely (l. 721-730).

In summary, I believe that the terms “quantitative” (lines 151 and 722) and “unification” (lines 60-61, 722) are not applicable here and that the authors should more clearly indicate the approximaitons and hypotheses they consider, as well as the differences with the experimental studies they cite. They also should perform additional analyses to compare their results with the experimental results in [17].

As mentioned above, we nuanced the formulations. We now say that it is a step towards unification, since it does unify a large set of data that were not compared before. Quantitative agreement with various sources of data remains a target.

Additional comments (more or less important):
- The authors often use the term “regulation” where “impact/effect” would be more appropriate. For instance, “Regulation by topological domain size” (line 507) suggets that cells can regulate this aspect, which is discutable. The authors should revise the text accordingly.

Agreed regarding the domain size. After consideration, we decided to keep the word “regulation” for topoisomerases, and mention in the text that the use of this word (involving an evolutionarily selected biological control mechanism) is still a hypothesis (l. 767-769). We renamed the sections on “Regulation by topological domain size” to “Impact of topological domain size”, and adjusted the text accordingly.

- The first sentence of the articles says “SC has been proposed as a new means to regulate transcription independent of regulatory protein binding [1]”. I see two issues here. First, I do not think that [1] proposes this. Second, this is not new (as explained in [1]).

We apologize for the wrong referencing. We removed the word “new” and replaced the reference that discusses this hypothesis (l. 81-83).

- Line 91 : I would add a work where they experimentally demonstrate this effect, e.g. the work by Leng et al. 10.1073/pnas.1109854108.

Done.

- Line 96-97 : I do not understand why [3] is cited here. I would cite at least Menzel and Gellert (1983) 10.1016/0092-8674(83)90140-x

Agreed.

- Line 101: related to te citation [8]: more recent reviews on topoisomerases exist and bring novel information not discussed in [8]. See for instance 10.1002/bies.202000286 and 10.1042/BST20240089

We now reference these two review articles.

- Line 107 : “While SC may serve as a new, important transcriptional regulator” : see above about the term “new”

Removed.

- Line 135-136 (and before): It is somewhat curious that [17] is not mentioned when introducing the single-gene approach. To my knowledge, [17] is the only experimental work that studies the impact of domain size on the expression rate of a single gene, exactly what is discussed in the proposed ms.

That study is cited at many locations since it indeed deals with questions closely related to this manuscript. As requested, it is now cited here too.

- Line 158: “Our model broadly follows an approach used in several previous works including ours [12, 17, 23-25]”. I see two problemes here: the lack of clear presentation of previous models (see general comments) and the mixing of models of different nature. For instance, the model used by the authors is directly related to [17] and [23]. The work [12] is of different nature as it does not consider stalling.

To address this issue, we introduced a presentation of previous models (see first request above). We find it delicate to define models of different “natures”, since there have been multiple models of this process that include different elements and mechanisms, and therefore result in different behaviors – some include stalling but differ by other aspects (see new Table S1). Considering that the sentence is nuanced by the adverb “broadly”, we think it fair to cite here a list of stochastic 1D models that deal with the reciprocal influence of supercoiling and bacterial transcription, even if they differ in the mechanisms they consider (some of these models may be missing). The reviewer is of course free to disagree on that point.

- Line 163: if the authors want to use the terminology “improvements”, they should also consider “deteriorations”. Namely, with respect to [17], i) they do not consider different stages for the initiation, which have been shown in [17] to be necessary to explain variations between promoters senstivty to the upstream distances, ii) they do not consider the specific activities of topoisomerases (see above). With respect to [23], they do not consider the pssibility for the RNAP to rotate.

We agree that the differences were not highlighted enough (also see answer to reviewer 1 regarding rotation), and that the word was ill chosen. We now rather use the word “simplification” than “deterioration”, and correspondingly replaced “improvements” by “additions”, related to the different objectives of the two mentioned studies. We recognize these simplifications in lines 158-160.

- Line 166: the work by Ashley et al. could be added (10.1093/nar/gkx649)

We added this work, which is indeed related, although the most important case for our simulations is negatively rather than positively supercoiled DNA.

- Line 177: a reference for plectoneme hopping could be added

We now cite Loenhout 2012, which measured the diffusion of supercoils, including by plectoneme hopping.

- Line 195: I would add that TopoI binds single-stranded DNA.

We now specify that gyrase binds dsDNA and topoI binds ssDNA.

- Line 217: a reference for the footprinting length of an elongationg RNAP could be added

We now cite Gardner 1990, which characterized the length of DNA protected from exonuclease by RNAP.

- Line 228: this maximal speed of RNAP is for minimal growing conditions. This should be added.

We now specify these conditions.

- Line 282: “TopoI” instead of “gyrase”

We appreciate the review noticing this typo and have corrected it.

- Line 285: the choice of $\sigma_T=\sigma_i=-0.04$ is discutable (and, hence, should be discussed) for at least two reasons: i) there is experimental work suggesting $\sigma_T=-0.05$ (10.1074/jbc.275.11.8103) and ii) differences between $\sigma_T$ and $\sigma_i$ have been shown to lead to qualiative different behaviors depending of the exact value of $\sigma_i$ [17]

We agree that this question deserves a notice to the reader, and added a sentence to discuss the implications of these parameter values in the Methods paragraph (l. 290-293). We agree that variations in these values could lead to differences in behavior. However, (1) the value of $\sigma_i$ is known to depend (quite strongly) on the promoter sequence, a feature that we disregard; (2) as far as we know, there is any clear measurement of a response function between topoI activity and local supercoiling. We added a reference to the study mentioned by the reviewer, but it is centered on topoIV and gyrase, not topoI, and only proposes an approximate value of -0.05 based on global supercoiling levels measured in different topoisomerase mutant strains, far from any measurement required for a direct parameterization. Our activation function (see Fig. 1) increases continuously between 0.03 and 0.05, and matches that used for transcription initiation. Altogether, we think the reviewer is right that this is an important point to mention, but will need more accurate data to obtain a definitive conclusion.

- Line 227 and page 18: it looks that the simulations require to introduce a (sufficiently negative) $\sigma_{start}$. What is the in vivo meaning of this value, knowing that by coherence, $\sigma_{start}$ should be given by the activity of topoisomerases (and hence, should not be necessary)? This should be discussed.

We added a remark on that point in Materials and Methods (Modeling method overview, l. 226). We are not sure what sentence gives the impression that the choice of $\sigma_{start}$ is important. It is arbitrary, and the steady-state value (after some initial simulation time in each run) is indeed set by topoisomerases – we just choose a reasonable initial value to keep this initial phase short.

- Line 296: I do not see how the authors conclude the value of $\rho_G=4$ from [36]. This should be explained. More importantly, the experimental work of Ashley et al. (10.1093/nar/gkx649) suggests a scenario in which gyrase activity involves a very high processivity intersperced with long periods of pauses, which differs from the scenario proposed by the authors. This should be discussed.

We found the value $\rho_G=4$ by a comparison from Figure 1d of Gore 2006 with a geometric distribution. We clarify this in the methods (l. 303).

- Results of Fig. 2: this suggests a scenario different from what has been proposed in [13]. This could be discussed.

This is a good point that we had missed during the writing process. We have included a new Discussion paragraph (l. 756-765).

- Line 507: “Regulation by topological domain size” => “Impact of …” (see above)

This change has been made, as detailed above.

- lines 508-510: this is really curious not to cite [17], here. Also, I do not see the relevancy of [48] here.

We have removed [48] and inserted reference [17].

- lines 553-555: I note that there is nothing new here with respct to [17].

We appreciate the feedback. We now note that our results are consistent with [17] (line 554).

- lines 580-582: it is not clear why the authors mention this since the Fano factor is a classical quantity to assess variability in gene expression.

We believe it was worth exploring the statistics that give us the clearest information on the variation within our model (this is related to reviewer 1’s question about why we didn’t use the coefficient of variation). While we choose to focus our analysis on the Fano factor, we think that including the coefficient of variation clarifies why we make this decision.

- line 620: “a moderate promoter (ki = 0.05 s-1) with intermediate barriers (D = 10 kb). This promoter strength and domain size (green triangles in Fig. 5A) are representative of many bacterial genes” : this is discutable. Indeed, it has been shown that transcription units, even those that are weakly expressed, often need to be considered within broader domains in which transcription is coordinated (when expressed) — see, for example, 10.1371/journal.pone.0155740. The idea that barriers 10 kb away from isolated genes (with in mind the size of topological domains in E. coli [43]) is a typical sitjuation is therefore hypothetical to me.

We agree that the wording was overstated. The presence, stability and localization of topological barriers in the E. coli chromosome remains quite elusive. We nuanced the sentence significantly (now l. 612-616).

- In the scenario 2 (lines ~ 640), it should be explained how the gene goes back to the on-state.

The gene returns to the on state because, despite a SC level that is unfavorable to initiation, due to the stochastic nature of our model occasionally an RNAP will initiate in spite of the supercoiling level. We add a sentence to the manuscript clarifying that rare initiation events are still able to occur from within the “off” state (l. 628-632).

- I note that the timescale of the off-state in scenario 2 is on the order of 10 minutes, in accord with experimental data (10.1038/ng.821). This could be mentionned.

We thank the reviewer for this good point and now cite this article (l. ~634-635).

- Line 665: in [17], there is no transcriptomics data

We apologize for the accidental inclusion of this reference and have removed it.

- lines 711 to 715: I do not understand why [50] supports the author’s statement. The work [50]concludes that there are approximately 300 gryases constantly bound along the chromosome, which is sufficient to relax supercoiling constraints in approximately the same number of supercoiled domains (see [43]). Meaning that there is in principle enough DNA gyrase ready to act on lowly expressed genes, contrary to what the authors state.

We corrected this sentence, where the reference was indeed misplaced and the wording was too definitive. We agree that, if the 300 gyrase molecules were perfectly evenly distributed among large topological domains where positive supercoils diffuse along the whole domain (in both directions around strongly expressed genes, which is not the prediction of the twin-supercoiled domain model), this number of molecules would be enough to relax most of the chromosome. We might disagree here, but we favor an alternate explanation where most genes do not need any gyrase to be transcribed, and the few hundreds of molecules might actually serve for a few hundreds genes. We corrected the location of the reference and rephrased the sentence as a hypothetical scenario.

- Line 732: [17] does not show that “stronger genes are relatively repressed by TopoI inhibition”

We have removed this reference.

- Line 766: “Boulas provides both experimental and computational evidence that transcription is mostly sensitive to the upstream barrier distance” : there is no computational evidence in [17]. The model is adjusted to fit the experimental evidence.

We rephrase this sentence to clarify that the experimental evidence regarding the upstream sensitivity was used to tune the computational model, rather than the computational model providing evidence for the upstream sensitivity.
